# Cystatin C: A Candidate Biomarker for Amyotrophic Lateral Sclerosis

**DOI:** 10.1371/journal.pone.0015133

**Published:** 2010-12-09

**Authors:** Meghan E. Wilson, Imene Boumaza, David Lacomis, Robert Bowser

**Affiliations:** 1 Department of Pathology, University of Pittsburgh School of Medicine, Pittsburgh, Pennsylvania, United States of America; 2 Department of Neurology, University of Pittsburgh School of Medicine, Pittsburgh, Pennsylvania, United States of America; Brigham and Women's Hospital, Harvard Medical School, United States of America

## Abstract

Amyotrophic lateral sclerosis (ALS) is a fatal neurologic disease characterized by progressive motor neuron degeneration. Clinical disease management is hindered by both a lengthy diagnostic process and the absence of effective treatments. Reliable panels of diagnostic, surrogate, and prognostic biomarkers are needed to accelerate disease diagnosis and expedite drug development. The cysteine protease inhibitor cystatin C has recently gained interest as a candidate diagnostic biomarker for ALS, but further studies are required to fully characterize its biomarker utility. We used quantitative enzyme-linked immunosorbent assay (ELISA) to assess initial and longitudinal cerebrospinal fluid (CSF) and plasma cystatin C levels in 104 ALS patients and controls. Cystatin C levels in ALS patients were significantly elevated in plasma and reduced in CSF compared to healthy controls, but did not differ significantly from neurologic disease controls. In addition, the direction of longitudinal change in CSF cystatin C levels correlated to the rate of ALS disease progression, and initial CSF cystatin C levels were predictive of patient survival, suggesting that cystatin C may function as a surrogate marker of disease progression and survival. These data verify prior results for reduced cystatin C levels in the CSF of ALS patients, identify increased cystatin C levels in the plasma of ALS patients, and reveal correlations between CSF cystatin C levels to both ALS disease progression and patient survival.

## Introduction

Amyotrophic lateral sclerosis (ALS) is a fatal neuromuscular disease that affects approximately 1.5 to 2.5 per 100,000 individuals of all races and ethnicities throughout the world [1]. ALS patients typically undergo rapid disease progression, though a subset exhibits slow progression and may live over a decade from symptom onset [2], [3]. Unfortunately, there is only one drug currently approved by the FDA to treat ALS, and this therapy increases life span by just two to three months on average [4]. Clinical disease management is also hindered by an often lengthy diagnostic process based predominately on clinical criteria [5]. As new drugs that slow or arrest disease progression become available, early initiation of treatment will become paramount. For this reason, diagnostic biomarkers for ALS must be identified and validated to maximize treatment efficacy for future patients. Several individual panels of CSF proteins have shown promise as candidate biomarkers, but none have been fully validated or integrated into clinical practice [6], [7], [8].

Biomarkers also hold promise to monitor disease progression and to stratify patient populations for use in clinical trials. One reason new drug therapies have not been successfully translated from ALS model systems to humans is ALS disease heterogeneity [9]. Biomarkers that monitor disease progression would aid in the design and execution of human clinical trials and would provide novel targets for future drug therapies; prognostic biomarkers that predict patient survival would also aid in the design of clinical trials. While there are several validated demographic and clinical prognostic factors for ALS, disease prognosis cannot currently be predicted with high accuracy within individual patients [10]. Ultimately, surrogate biomarkers of disease progression would provide a means to more rapidly monitor drug efficacy in clinical trials [5], [9], [11], [12]. Therefore, the search for biomarkers that fit these functional characteristics represents a key challenge toward improving drug therapies and clinical management for ALS.

One protein that has shown potential for ALS diagnostic utility is cystatin C, a widely expressed cysteine protease inhibitor that is approximately five times more abundant in CSF than in plasma [13]. Cystatin C is processed through the secretory pathway, and, in its active monomeric form, inhibits a wide variety of cysteine proteases including cathepsins B, H, L, and S, calpains and caspases [14]. Cystatin C is also linked to ALS histopathologically, as it is one of only two known proteins that localize to Bunina bodies, which are small intraneuronal inclusions specific to ALS [15].

Two prior surface-enhanced laser desorption/ionization time of flight mass spectrometry (SELDI-TOF-MS) studies reported significant decreases in CSF cystatin C levels in ALS patients relative to healthy controls [6] and mixed healthy/neurologic disease controls [7]. A recent study using small numbers of test subjects reported a significant reduction in CSF cystatin C concentration in ALS patients relative to individuals with polyneuropathy, as measured by ELISA [16]. While these prior studies are encouraging, a larger study with a more comprehensive group of ALS-mimic disease controls is required in order to verify prior results and determine if CSF cystatin C levels represent a candidate diagnostic biomarker for ALS.

The objective of this study was to use quantitative ELISA to further evaluate the utility of cystatin C as a biomarker for ALS using a large subject population. Our subject group size was based on power analysis of previously published mass spectrometry reports on cystatin C in ALS [6], [7]. We evaluated cystatin C in both CSF and plasma as a candidate diagnostic biomarker, and correlated levels to individual ALS patient survival and disease progression. We verified that cystatin C protein levels are reduced in the CSF of ALS patients and discovered that cystatin C levels are increased in the plasma of ALS patients. However, cystatin C levels in either biofluid were not highly predictive of ALS. We also determined that CSF cystatin C levels correlate to ALS patient survival, and change during disease progression.

## Results

We collected longitudinal CSF and plasma samples from 104 ALS and control subjects (Table 1) and evaluated the absolute cystatin C concentrations by ELISA and the total sample protein concentrations by BCA protein assay. We then assessed the biomarker utility of two separate measures of cystatin C: (1) the absolute cystatin C concentrations or “total cystatin C” and (2) the “percent cystatin C,” in which the absolute cystatin C concentrations were normalized to the total sample protein concentrations to determine the percent of total biofluid protein accounted for by cystatin C. We also collected several clinical measures of disease progression at the time of each biofluid draw (see methods).

**Table 1:** Clinical characteristics of all study participants.

	ALS (n = 44)	All Disease Controls (n = 25)	Mimic Disease Controls (n = 9)	Healthy controls (n = 35)
**Sex (male/female)**	31/13	13/12	7/2	13/22
**Age at first draw ± SD (years)**	54.8±13.5	47.9±15.4	57.2±11.8	46.8±15.6
**Relevant subgroups**	35 limb onset, 5 bulbar onset, 4 mixed/other onset	9 ALS mimics, 6 MS, 10 other	2 PLS, 2 CIDP, 2 PMA, 1 SA, 1 small fiber neuropathy, 1 idiopathic sensorimotor polyneuropathy	NA

MS  =  multiple sclerosis; PLS  =  primary lateral sclerosis; CIDP  =  chronic inflammatory demyelinating polyneuropathy; PMA  =  progressive muscular atrophy; SA  =  spinocerebellar ataxia; NA  =  not applicable.

### Diagnostic biomarker assessment

In order to assess the diagnostic utility of cystatin C, we first compared the mean first-draw cystatin C levels among ALS patients, neurologic disease controls, and healthy controls. A generalized linear model was used to estimate the mean total and percent cystatin C for each diagnostic category, with both gender and age included as co-factors in the model. This statistical design controls for between-group differences in each co-factor when generating estimated means. Therefore, the differences in age and gender among our diagnostic groups should not have affected our results, even in the case that cystatin C varies with these factors. We found that the estimated means for both measures of cystatin C were lower in ALS patients than in disease controls and healthy controls, similar to prior studies (Table 2). However, a test of the model's main effects revealed that only percent cystatin C differed significantly by disease diagnosis, while total cystatin C levels were not significantly different across diagnostic groups. Neither measure of cystatin C differed significantly by age or gender. In a post-hoc pairwise comparison of diagnostic groups, percent cystatin C was found to be significantly lower in CSF of both ALS patients and disease controls relative to healthy controls, but there was no statistical difference between cystatin C levels in ALS patients and disease controls.

**Table 2:** CSF main group results for total and percent cystatin C.

	Total Cystatin C	Percent Cystatin C
	Mean (ug/ml) ± S.E.M.	Mean (%)± S.E.M.
**ALS (n = 44)**	3.32±.19	0.40±0.02
**DC (n = 25)**	3.61±.26	0.45±0.03
**HC (n = 35)**	4.00±.25	0.54±0.03
**Significance of Model Main Effects (p-values)**		
**Diagnosis**	0.109	0.002*
**Gender**	0.400	0.740
**Age at Draw**	0.367	0.672
**Pairwise Comparisons by Diagnosis (p-values)**		
**ALS vs. DC**	0.384	0.259
**ALS vs. HC**	0.038*	0.001*
**DC vs. HC**	0.277	0.034*

Percent cystatin C differed significantly by diagnostic category and was significantly reduced in both ALS patients and disease controls relative to healthy controls. ALS  =  all ALS patients; DC  =  all neurologic disease controls; HC  =  healthy controls. Asterisks indicate statistical significance at p<0.05.

Next, we repeated these statistical analyses using data from specific patient subgroups, in order to determine if either measure of CSF cystatin C can be used to differentiate ALS patients from disease controls in specific patient subpopulations. First, we created a subcategory of disease controls comprised of patients with neurologic diseases that more closely resemble ALS at presentation. Using this group of ALS mimics in our statistical model, the patterns of overall and between-group statistical differences remained the same (data not shown). However, the *p*-values were reduced for the ALS vs. mimic disease control subgroup comparison (Table 3, first row) relative to the ALS vs. all disease control subgroup comparison (Table 2, “ALS vs. DC”) suggesting a stronger trend toward statistical significance when cystatin C is used to differentiate ALS patients from this more clinically-relevant control group.

**Table 3:** CSF subgroup results for total and percent cystatin C.

	N	Mean Total Cystatin C (ug/ml) ± S.E.M.	Significance of Pairwise Difference (p-values)	Mean Perecent Cystatin C ± S.E.M.	Significance of Pairwise Difference (p-values)
**ALS vs mimic DC**	44/9	3.35±0.19 vs 3.99±0.48	0.212	0.40±0.02 vs 0.49±0.06	0.129
**ALS-L vs mimic DC**	35/9	3.33±.021 vs 4.00±0.49	0.196	0.40±0.02 vs 0.49±0.06	0.098
**ALS>1yr vs mimic DC**	29/9	3.24±0.22 vs 3.99±0.48	0.151	0.39±0.03 vs 0.49±0.06	0.093

The diagnostic potential of both measures of cystatin C, as implied by the pair-wise difference p-values, was improved when comparing ALS to mimic DC rather than all DC (top row vs. Table 2). Additionally, the diagnostic potential vs. mimic DC was higher for two ALS subgroups, ALS-L and ALS>1yr, than for all ALS patients combined. ALS-L  =  limb-onset ALS; ALS>1yr  =  patients with first biofluid draw occurring more than one year following symptom onset.

We next compared two ALS subgroups to the disease mimic group. Limb-onset ALS (ALS-L) and ALS patients greater than one year from symptom onset both exhibited reduced levels of cystatin C in the CSF when compared to disease mimics (Table 3), with improved *p*-values when compared to the analysis including all ALS patients. However, the pair-wise comparisons between these ALS subgroups and mimic disease controls still fell short of statistical significance.

Finally, we calculated the diagnostic sensitivity and specificity for several cutoff concentration values of CSF cystatin C. Total cystatin C concentration measurements were found to have better diagnostic parameters than percent cystatin C values. A cutoff value of 2.20 µg/ml identified a small subset of ALS patients (sensitivity: 23%) with relatively high specificity (88% vs. all study controls, 100% vs. mimic disease controls). A cutoff value of 2.70 µg/ml identified a modest subset of ALS patients (sensitivity: 32%) while maintaining high specificity versus controls (specificity: 78% vs. all study controls, 100% vs. mimic disease controls). A less conservative cutoff value of 3.50 µg/ml identified a majority of ALS patients (sensitivity: 52%), but demonstrated lower specificity (specificity: 52% vs. all study controls, 89% vs. mimic disease controls).

As noted above, cystatin C was previously reported to be significantly reduced in the CSF of ALS patients using mass spectrometry-based proteomics, but the between-group differences based on our ELISA data were less robust. To explore the relationship between CSF cystatin C levels measured by these two techniques, we compared our ELISA results with SELDI-TOF-MS data for the same CSF samples. We found significant, positive correlations between the 13.3 kDa SELDI-TOF-MS mass peak intensity for cystatin C and both total cystatin C and percent cystatin C protein levels as measured by ELISA (p = 0.002 and p<0.001, respectively; Figure S1). However, the correlation coefficients (Spearman r = 0.443 and 0.595, respectively) suggest that these techniques may be differentially sensitive to various modified forms of native cystatin C.

We repeated the group analysis for the diagnostic utility of cystatin C in plasma, and both measures of cystatin C varied significantly by diagnosis and age, but not by gender (Table 4). Post-hoc analyses revealed that total and percent cystatin C were significantly increased in both ALS patients and disease controls relative to healthy controls. However, there were no differences in cystatin C levels between ALS patients and disease controls. Identical trends were observed for all subgroup analyses of cystatin C levels in plasma (data not shown).

**Table 4:** Plasma main group results for total and percent cystatin C.

	Total Cystatin C	Perecent Cystatin C
	Mean (ug/ml) ± S.E.M.	Mean (%)± S.E.M.
**ALS (n = 43)**	0.818±0.024	1.06×10^−3^±3.36×10^−5^
**DC (n = 11)**	0.861±0.048	1.12×10^−3^±6.78×10^−5^
**HC (n = 31)**	0.705±0.023	0.89×10^−3^±3.17×10^−5^
**Significance of Model Main Effects (p-values)**		
**Diagnosis**	0.001*	<0.001*
**Gender**	0.457	0.293
**Age at Draw**	0.004*	0.003*
**Pairwise Comparisons by Diagnosis (p-values)**		
**ALS vs. DC**	0.442	0.419
**ALS vs. HC**	0.001*	<0.001*
**DC vs. HC**	0.004*	0.002*

Both measures of cystatin C differed significantly by age at draw and by diagnostic category. Cystatin C levels were significantly elevated in ALS patients and disease controls relative to healthy controls but there were no differences in cystatin C levels between ALS patients and disease controls. Asterisks indicate statistical significance at p<0.05.

To further characterize the relationship between CSF and plasma cystatin C levels, we assessed the correlation between CSF and plasma cystatin C levels for individual subjects. The results indicated that there is no correlation between total cystatin C concentrations (Spearman r = 0.055; p = 0.626) or percent cystatin C levels (Spearman r = −0.076; p = 0.501) in CSF and plasma samples drawn from individual patients on the same day (Figure S2). The absence of a relationship between CSF and plasma cystatin C levels suggests that this protein is independently regulated in both biofluid pools, and that plasma cystatin C is unlikely to be directly influenced by CSF levels.

### Cystatin C as a biomarker for disease progression

**Figure 1:**
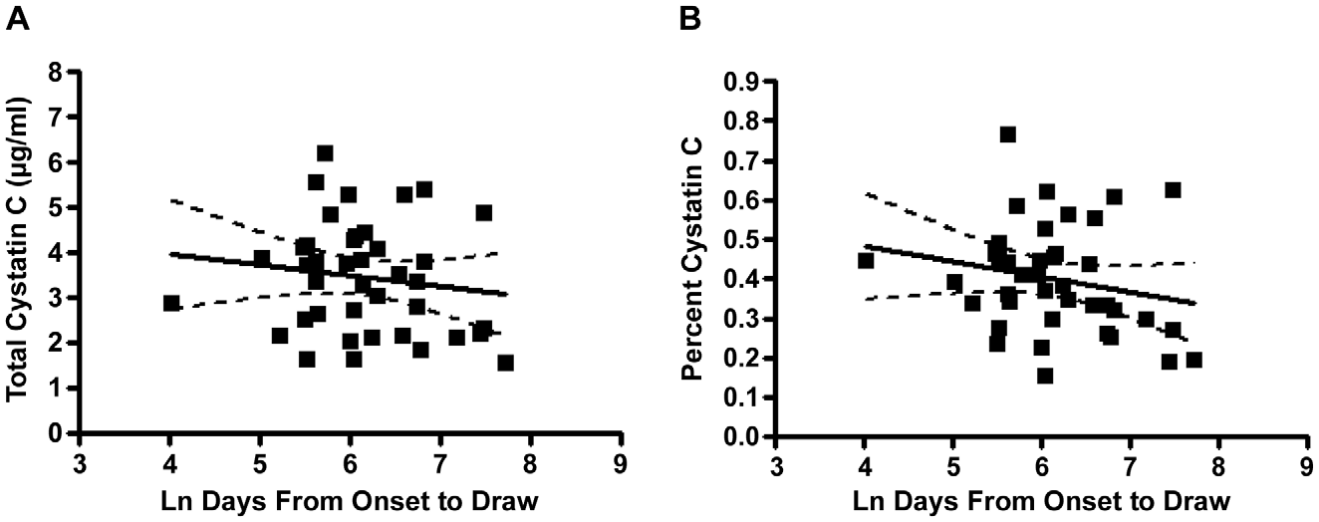
Linear regressions for CSF cystatin C levels vs. time from symptom onset. The slope of the best-fit lines (solid) for both total (**A**) and percent (**B**) cystatin C did not significantly differ from zero (*p* = 0.368 and *p* = 0.193, respectively). Dashed line = 95% confidence interval for best-fit line.

We next examined whether cystatin C levels change over time in ALS patients, and if these changes are associated with clinical disease progression. We compiled the first CSF draws for each of the ALS patients in our study and carried out linear regressions comparing both total and percent cystatin C with the time from symptom onset. Similar to a prior study [16], we found no statistically significant linear relationship between these variables in our data set (Figure 1). However, we did observe a slight trend toward a reduction in cystatin C levels over time from symptom onset, particularly for percent cystatin C (Figure 1B).

Next, we collected longitudinal CSF samples from ALS patients and assessed the effect of time on cystatin C levels using a statistical model for repeated measures. This experimental design controls for individual differences in baseline cystatin C levels, but not for individual differences in rate of disease progression. We collected at least three longitudinal CSF draws from 15 ALS patients over a 1–2 year time period for each patient. When all 15 values were averaged for each time point, we did not observe a significant change in cystatin C levels over time (Table 5, “All Patients”).

**Table 5:** Repeated measures tests for the change in total cystatin C concentration over time.

Total Cystatin C (µg/ml)	Draw 1 Mean ± S.E.M.	Draw 2 Mean ± S.E.M.	Draw 3 Mean ± S.E.M.	Trend Over Time	Change Over Time (p-values)	Time*Progression Speed (p-values)
**All Patients (n = 15)**	3.54±0.27	3.64±0.28	3.62±0.29	flat	0.663	N/A
**Fast Progressors (n = 6)**	4.11±0.30	4.02±0.34	3.82±0.36	↓	0.333	0.032*
**Slow Progressors (n = 9)**	3.17±0.20	3.39±0.23	3.49±0.26	↑↑	0.058	

There were no significant longitudinal changes in CSF cystatin C concentration in ALS patients as a combined group, but fast progressors showed a moderate longitudinal decrease and slow progressors showed a moderate longitudinal increase. There was a significant interaction between the change in cystatin C concentration over time and patient progression speed (fast versus slow progressors) as listed in Time.

*Progression speed column (*p* = 0.032). Asterisk indicates statistical significance at *p*<0.05.

To control for individual differences in disease progression speed, we separated ALS patients into two groups: fast progressors, who demonstrated a rapid clinical decline during the study period, and slow progressors, whose clinical decline was slower than average (see methods section). When the rate of disease progression was included as a factor in the statistical model, we found a significant interaction between the effects of time and progression speed for total cystatin C measurements (Table 5, Time*Progression Speed column), indicating that longitudinal changes in cystatin C concentration follow different patterns in the two patient subpopulations. In order to determine the direction and significance level of the longitudinal subgroup changes responsible for this interaction, we applied the repeated measures test individually to each patient subgroup. Fast progressors exhibited a subtle, non-significant decrease in cystatin C levels over time. In contrast, slow progressors exhibited a trend of increasing cystatin C levels over time (p = 0.058, Table 5), which likely accounts for the majority of the time/progression speed interaction. Similar trends were observed for percent cystatin C measurements. For comparison, we also assessed the longitudinal change in CSF cystatin C levels in 10 healthy controls, each with two CSF samples drawn 1.5–2 years apart. A repeated measures t-test revealed a modest increase in total cystatin C concentration over time in these healthy controls but no longitudinal change in percent cystatin C levels (data not shown).

### Correlation of cystatin C to survival

Finally, we assessed the relationship between first-draw cystatin C levels (in CSF and plasma) and patient survival time. Neither measure of plasma cystatin C showed a correlation with subsequent survival time (total cystatin C: Spearman r = −0.17, *p* = 0.537), but both measures of CSF cystatin C levels showed a direct correlation, with the results for total cystatin C almost reaching statistical significance (total cystatin C: Spearman r = 0.465, *p* = 0.052). These findings suggest that cystatin C levels in CSF but not plasma may be useful as prognostic indicators of patient survival time.

**Figure 2:**
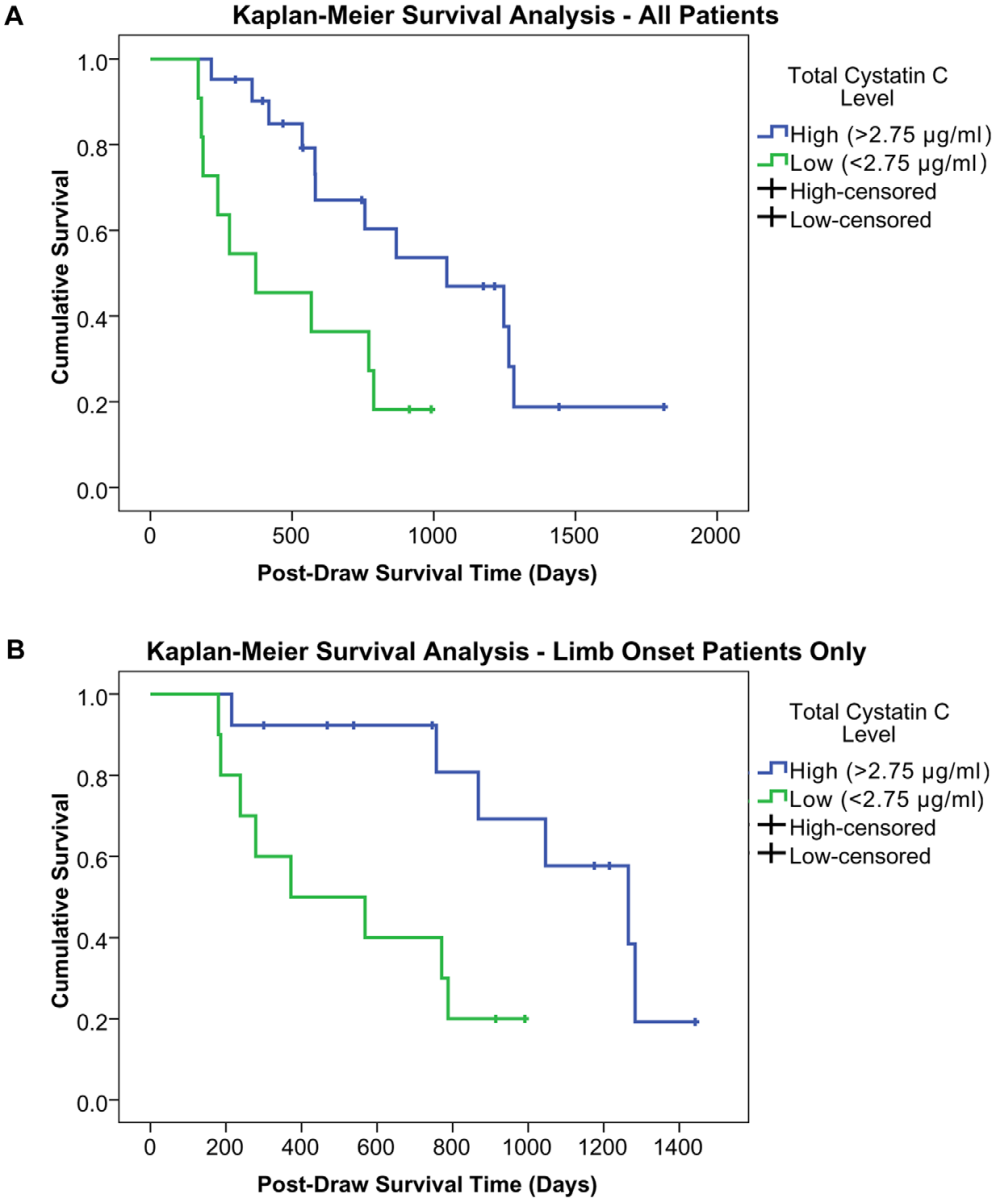
Kaplan-Meier survival curves. For all ALS patients (**A**), survival was significantly longer (*p*<0.014) in patients with high cystatin C levels (n = 21) than in patients with low cystatin C levels (n = 11). For patients with limb onset ALS (**B**), the same trend was observed, but with a larger survival difference (*p*<0.010) between patients with high (n = 13) and low (n = 10) cystatin C levels.

We further explored this correlation by generating Kaplan-Meier survival curves for total CSF cystatin C measurements. For these analyses, patients were sorted into high- and low-cystatin C groups according to their first-draw cystatin C levels. Qualitative data assessment revealed that short survival times were most strongly associated with the lowest cystatin C levels and, for this reason, we selected a cut-off value of 2.75 µg/ml to separate the ALS patients into a smaller low cystatin C group (n = 11) and a larger high cystatin C group (n = 21). This analysis revealed significantly longer patient survival in the high cystatin C group than in the low cystatin C group (Figure 2A). Next, because the ALS disease course and average survival time differ significantly between limb-onset ALS and bulbar-onset ALS, we repeated these statistical tests with exclusively limb-onset patients. Within this population, the between-group difference in post-draw survival time became even more striking (Figure 2B), further reinforcing our finding that ALS patients with low CSF cystatin C levels exhibit reduced survival times relative to patients with average to high CSF cystatin C levels. Similar results were obtained using percent cystatin C measurements.

## Discussion

The present study represents a comprehensive evaluation of cystatin C as a candidate biomarker in ALS, and is unique in its assessment of two distinct biofluids (CSF and blood plasma), two different measurements of the protein of interest (total concentration and percent of total protein), and longitudinally collected CSF samples. Prior studies using SELDI-TOF-MS found significantly lower cystatin C abundance in the CSF of ALS patients relative to healthy controls [6] and mixed healthy/neurologic disease controls [7]. These findings were validated by ELISA and immunoblot, respectively. In our current ELISA study for diagnostic utility using a much larger number of total subjects, we also found a significant reduction in cystatin C levels in the CSF of ALS patients relative to healthy controls, but the magnitude of this difference was less robust than in the previous reports. This discrepancy may have resulted from the use of different experimental techniques, as SELDI-TOF-MS recognizes discrete mass-to-charge forms of cystatin C, whereas ELISA may recognize multiple modified or cleaved forms of cystatin C depending upon the antibodies used for capture and detection. Regarding the comparison of these two techniques, we found a significant, positive correlation but a low correlation coefficient between our CSF ELISA data and SELDI-TOF-MS data for the same samples (Figure S1). This finding suggests that these techniques are sensitive to different, but possibly overlapping, ranges of native cystatin C isoforms, and may provide differential, and perhaps complementary, utility in detecting cystatin C for biomarker assessment.

To be clinically useful as a diagnostic biomarker, cystatin C must also be able to differentiate between ALS patients and individuals with neurologic diseases that closely resemble ALS, or ALS “mimic diseases.” A recent study reported a significant reduction in CSF cystatin C levels in ALS patients relative to polyneuropathy patients [16]. In our ELISA analysis, cystatin C was reduced in the CSF of ALS patients relative to all DC combined and, to a greater degree, relative to a mimic disease control group that included a variety of ALS mimics (Table 3), but neither difference reached statistical significance. Because these between-group differences were smaller than we expected based on previous mass spectrometry data [17], we conducted a new power analysis using our experimentally-derived group means and standard deviations. This analysis revealed that our study was adequately powered for comparing percent cystatin C between ALS and HC (a significant difference was found), and underpowered for comparing total cystatin C between ALS and HC (main group effects missed significance, but the pairwise comparison was significant), and for comparing ALS with DC for both measures of cystatin C (no significant differences were identified). The observed reductions in both total and percent cystatin C in ALS patients relative to DC may reflect actual differences in clinical cystatin C levels, but a total study enrollment of 1020 and 675 patients (for total and percent cystatin C, respectively) would be required to confirm statistical significance with 80% power and 95% confidence. Interestingly, the between-group differences and trend towards significance improved when comparing limb-onset ALS patients or ALS patients with disease course greater than 1 year from symptom onset to the ALS mimics (Table 3). Additionally, we found that the total cystatin C concentration measurement generated superior diagnostic accuracy, indicating that this may be the more efficacious measure of cystatin C. An assessment of the diagnostic parameters of CSF cystatin C concentration revealed that the sensitivity of cystatin C for differentiating ALS patients from disease controls is low for all cutoff values but it displays high levels of specificity and, therefore, cystatin C can only identify a small subset of ALS patients. Together, these findings indicate that CSF cystatin C levels may differ between ALS patients and relevant disease control populations but cystatin C, by itself, has limited diagnostic utility. However, this protein could potentially improve the sensitivity and/or specificity of a diagnostic biomarker panel. Due to the heterogeneous nature of the ALS patient population, it is likely that a multiple biomarker panel will be required, as opposed to any single protein biomarker, in order to differentiate ALS from related disorders with adequate diagnostic certainty [18].

We also assessed the diagnostic utility of plasma cystatin C levels. Plasma cystatin C has been extensively characterized as a peripheral biomarker for kidney function and as a prognostic indicator of the risk of morbidity and mortality relating to cardiovascular disease [19], [20]. However, blood-borne levels of cystatin C have not been evaluated as a biomarker candidate for neurologic disorders. We found that plasma cystatin C levels are equivalently elevated in both ALS patients and disease controls relative to healthy controls, indicating that elevated plasma cystatin C is a nonspecific finding associated with neurologic disease states. Therefore, plasma cystatin C levels, as evaluated by ELISA, do not to have diagnostic utility for ALS. Furthermore, the absence of a relationship between cystatin C levels in concurrently-drawn CSF and plasma samples from individual patients in this study (Figure S2) suggests that this protein is independently regulated in each biofluid. Accordingly, plasma cystatin C levels are unlikely to be directly correlated with motor neuron degeneration in ALS, though elevated levels may correlate to peripheral metabolic or inflammatory abnormalities during ALS.

A recent study examined a single CSF draw per ALS patient, taken at varying times from symptom onset, to indirectly infer the average longitudinal change in cystatin C concentration in the group as a whole, and they reported that cystatin C levels do not change over time [16]. We completed a similar analysis and also found no evidence for a patterned directional change in CSF cystatin C levels over time in ALS patients (Figure 1). However, both heterogeneity in disease progression speed and individual variation in baseline cystatin C levels could mask significant trends in cystatin C change over the course of disease progression and, therefore, single-draw protein levels are unsuitable for a thorough assessment of longitudinal trends in cystatin C abundance.

We also examined longitudinal CSF data from multiple patients to more accurately assess the changes in cystatin C over time. We found that longitudinal cystatin C concentrations were relatively constant in ALS patients as a combined group. In contrast, the subgroup of patients with slow or absent clinical disease progression exhibited longitudinal increases in cystatin C concentration, and the subgroup with more typical, continuous clinical deterioration exhibited longitudinal decreases in total cystatin C. Interestingly, slow progressors often exhibited lower initial levels of CSF cystatin C than fast progressors (Table 5). Similar trends were also observed for percent cystatin C measurements, but statistical significance was not reached. These results indicate that CSF cystatin C levels in ALS patients change over time in a clinically-relevant manner and that increasing cystatin C concentration may be associated with slower disease progression. Conversely, rapid disease progression may be associated with a decrease in cystatin C concentration over time.

We also conducted an analysis to determine the relationship between longitudinal changes in CSF cystatin C levels and time-matched changes in three functional clinical measures of disease progression (ALSFRS-R, MMT, and FVC). However, no significant correlations were found (data not shown). This indicates that cystatin C levels may change independently of the clinical parameters used for monitoring disease progression. However, this finding does not eliminate the possibility that changes in CSF cystatin C levels correlate with more subtle biochemical changes associated with disease progression, as these may not be accurately reflected by overt functional measures of clinical disease status [9], [11]. Furthermore, the observed trend of increasing cystatin C levels in patients with slow rates of clinical deterioration may prove to be useful as an objective biomarker for monitoring drug effects in clinical trials.

We recently demonstrated a correlation between CSF cystatin C levels and patient survival by SELDI-TOF-MS [17]. In this study, we further verified a direct correlation between CSF cystatin C concentration and patient survival time, supporting the potential utility of this protein for prognostic applications. Subsequent Kaplan-Meier survival analyses for patient groups with CSF cystatin C concentrations above and below qualitatively selected cut-off values confirmed significantly longer survival times for patients in the higher cystatin C groups. Additionally, the prognostic capacity of CSF cystatin C was higher for limb-onset patients (Figure 2B) than for all patients combined (Figure 2A). This may have resulted from the confounding effects of combining patients with different sites of disease onset, as bulbar-onset ALS patients typically have shorter survival times than limb-onset patients [2], [3]. Unfortunately, there were inadequate numbers of bulbar-, trunk-, and/or dementia-onset patients to analyze these individual subgroups in this study, and further analyses are required to determine the prognostic capacity of cystatin C in these subgroups. Nonetheless, these results show that cystatin C is a candidate prognostic indicator of survival in ALS patients. Alternatively, cystatin C levels could contribute to the process of balancing prognostic variables among experimental groups as recommended to equalize drop-out rates and preserve the balancing effects of randomization in clinical trials [9]. Further work is required to more fully characterize the relationship between CSF cystatin C concentration and ALS patient survival, and to determine optimal cut-off values and procedures to stratify patients for prognostic purposes.

The results of this comprehensive biomarker assessment also have implications for the potential mechanistic involvement of cystatin C in the pathogenesis of ALS. The function of cystatin C within the CNS has not been extensively studied, but it appears to have both neurotoxic and neuroprotective properties [21], [22], [23], [24], though its effects specifically on motor neurons have not been reported. The majority of cystatin C in the CSF is produced by the choroid plexus [25], but it is unclear whether the apparent reductions in CSF levels in ALS patients are an independent etiological factor contributing to motor neuron degeneration, a downstream result of disease pathogenesis, or a compensatory response to ALS pathology. However, the association of higher cystatin C concentrations with longer patient survival and the association of increasing cystatin C levels with slower clinical progression both suggest that extracellular cystatin C may exhibit neuroprotective properties within the context of ALS. This would implicate any absolute or relative cystatin C deficiency in ALS as both a potential contributor to disease pathogenesis and a potential therapeutic target. Continuing work in our laboratory is focused on determining the effects of altered cystatin C concentration/activity on motor neurons *in vitro*, in order to clarify its potential mechanistic role in ALS pathogenesis.

In summary, we have completed a comprehensive evaluation of cystatin C as a candidate ALS biomarker, including assessments of two complementary measures of cystatin C in two distinct biofluids as well as examinations of both longitudinal CSF samples and patient survival data. Our findings indicate that cystatin C levels, as determined by ELISA, are increased in the plasma and decreased in the CSF of ALS patients relative to healthy controls. CSF cystatin C measurements may possess a more limited diagnostic capacity for ALS than previously proposed, but may still have the potential to improve the diagnostic parameters of a biomarker panel. Additionally, longitudinal changes in CSF cystatin C levels may be useful as a biomarker of fast versus slow rates of disease progression. Our data also demonstrate that CSF cystatin C concentration has prognostic utility in estimating patient survival time. Further validation studies are necessary to confirm these findings and ultimately determine if cystatin C measurements can be used to enhance clinical disease management and clinical trial design. Finally, the association of high or increasing cystatin C levels with slower disease progression and increased survival time suggests a potential neuroprotective role for this protein in the pathobiology of ALS.

## Materials and Methods

### Sample Collection and Ethics Statement

This study was approved by the institutional review board (IRB) at the University of Pittsburgh, and written informed consent was obtained from all participating subjects. ALS subjects were diagnosed by experienced neurologists specialized in motor neuron disease, using revised El Escorial criteria [26]. CSF and plasma samples were collected at the same office visit every four to six months from 44 ALS patients (2–8 draws), and either once or twice (1.5–2 years apart) from 35 non-neurologic healthy controls (HC) and 25 neurologic disease controls (DC). Our total enrollment of 104 patients provided adequate power for this study as a pre-study power analysis using projected effect sizes based on previous mass spectrometry findings [17] concluded that a total enrollment of 96 patients was required to identify pairwise differences between ALS patients and both HC and DC groups for both measures of cystatin C. We did not control for potential confounding variables such as socioeconomic status, nutrition, environmental exposures, etc. between diagnostic groups. The median time from symptom onset to first draw for ALS patients was 468 days. Clinical parameters used to monitor ALS disease progression included the rate of change in the revised ALS functional rating scale (ALSFRS-R), manual muscle strength tests (MMT), and forced vital capacity (FVC) [27], [28], [29].

The disease control group included six patients with multiple sclerosis, one with bilateral facial palsies, one with neurosarcoidosis, one with viral encephalitis, one with CNS lymphoma, one with brain metastases, one with pseudotumor cerebri, one with a seizure disorder, one with complicated migraine, one with paresthesis and possible myelopathy, one with a probable conversion disorder, and nine with neurologic diseases that can clinically resemble ALS at presentation. This ALS-mimic disease subgroup included two patients with primary lateral sclerosis (PLS), two with chronic inflammatory demyelinating polyneuropathy (CIDP), two with progressive muscular atrophy, one with spinocerebellar ataxia, one with small fiber neuropathy, and one with idiopathic sensorimotor polyneuropathy. CSF samples were obtained by lumbar puncture, immediately centrifuged at 450 g for five minutes at 4°C to remove cells and debris, aliquoted, and then frozen at −80°C. Intravenous blood samples were collected in EDTA containing tubes, inverted to mix, and centrifuged at 1,733 g for 10 min at 4°C. The plasma was decanted, aliquoted, and frozen at −80°C. CSF and plasma were aliquoted into small volumes for single use in experiments in order to eliminate any freeze/thaw effects. Samples of either biofluid were thawed on ice immediately prior to use.

### Cystatin C ELISA

CSF and plasma samples from individual patients were assigned to random 96-well plate positions, and evaluated in duplicate wells for each ELISA. All samples were independently assayed at least twice. For all experiments, we used a human cystatin C sandwich ELISA kit (Biovendor, Candler, NC), according to the manufacturer's instructions. Briefly, diluted CSF and plasma samples (1∶2000 and 1∶400, respectively in dilution buffer) were applied to antibody pre-coated ELISA plates for 30 min with gentle agitation. The wells were washed thoroughly and then the secondary antibody conjugate solution was applied for 30 min with gentle agitation. After a second wash, the 3,3′,5,5′ Tetramethylbenzidine substrate solution (Biovendor, Candler, NC) was applied for 10 min, color development was stopped with an acidic stop solution, and the optical density was measured at 450 nm using a plate reader. Total protein concentrations for each sample were calculated using a BCA protein assay (Pierce), according to the manufacturer's instructions.

### Statistics

#### Data Processing

For each ELISA plate, a standard curve was generated by plotting the logarithm of the cystatin C concentration against the logit log of the adjusted optical density (divided by a constant to produce a data range between zero and one, as required for the logit logarithm function). This procedure produced a linear standard curve, which was then used to calculate sample cystatin C concentration from sample optical density. The data points were averaged to determine the absolute cystatin C concentration, or “total cystatin C,” for each sample. Sample cystatin C concentrations were normalized to the sample total protein concentration to determine the percent of total biofluid protein accounted for by cystatin C, or “percent cystatin C.”

#### Assessment of Diagnostic Biomarker Utility

For the analyses of diagnostic utility, we included only the initial sample collected from each patient, representing the time point closest to symptom onset. Differences between group and subgroup means were identified using the SPSS generalized linear model, with diagnosis and sex as factors in the model and age at draw as a covariate. This model was subsequently used to calculate and compare the estimated marginal group means, in order to determine which pairwise differences among the levels of each factor were responsible for the significant main effects.

#### Assessment of Longitudinal Change in Cystatin C

The relationship between first-draw cystatin C levels and the length of time from symptom onset was assessed by linear regression using GraphPad Prism 5.0 software (GraphPad Software Inc, La Jolla, CA). For this analysis, the “time from symptom onset” data were transformed by the natural logarithm (Ln) in order to achieve normality as required by the selected statistical test.

The effect of time on longitudinal cystatin C levels in ALS patients with multiple biofluid draws was assessed with SPSS software, using the general linear model for repeated measures. The model was applied for all patients combined and for patient subgroups sorted by progression speed. Fast progressors were defined as patients exhibiting above average rates of ALSFRS decline (median: 0.77 units/month [30]) or MMT decline (mean: drop of 1%/month [29]), and slow progressors were defined as patients exhibiting smaller than average longitudinal decreases in both of these clinical progression measures.

The longitudinal relationship between cystatin C levels and clinical disease progression in individual patients was assessed by nonparametric correlation analysis (GraphPad Prism 5.0). The Spearman's rank correlation coefficient (r) was calculated and the permutation test was applied to determine if r was significantly different from zero.

#### Assessment of Prognostic Biomarker Utility

The relationship between first-draw cystatin C levels and post-draw survival time (for deceased patients only) was assessed by Spearman correlation analysis (GraphPad Prism 5.0). The prognostic utility of CSF cystatin C was further explored by generating Kaplan-Meier survival curves for patients falling above or below several cut-off values of cystatin C. SPSS software was used to calculate the p-values for differential survival time by three different methods: the Log Rank (Mantel-Cox) test, the Breslow (Generalized Wilcoxon) test, and the Tarone-Ware test. For all statistical analyses in this study, the significance level was set at p<0.05.

## Supporting Information

Figure S1 Correlation of ELISA-based cystatin C levels and SELDI-TOF-MS 13.3 kDa mass peak intensity levels by Spearman correlation analysis. Both total (A) and percent (B) cystatin C ELISA measurements correlated to the 13.3 kDa cystatin C mass peak.
(TIF)

Figure S2 Correlation analysis for cystatin C levels in CSF and plasma. There was no correlation between total cystatin C concentrations (A) (r = 0.055; p = 0.626) or percent cystatin C levels (B) (r = -0.076; p = 0.501) between CSF and plasma.
(TIF)
