# Supplementary figures and images for: Cystatin C: A Candidate Biomarker for Amyotrophic Lateral Sclerosis
Source: PLoS One. 2010 Dec 9;5(12):e15133. doi: 10.1371/journal.pone.0015133 (PMC3000338; doi:10.1371/journal.pone.0015133)

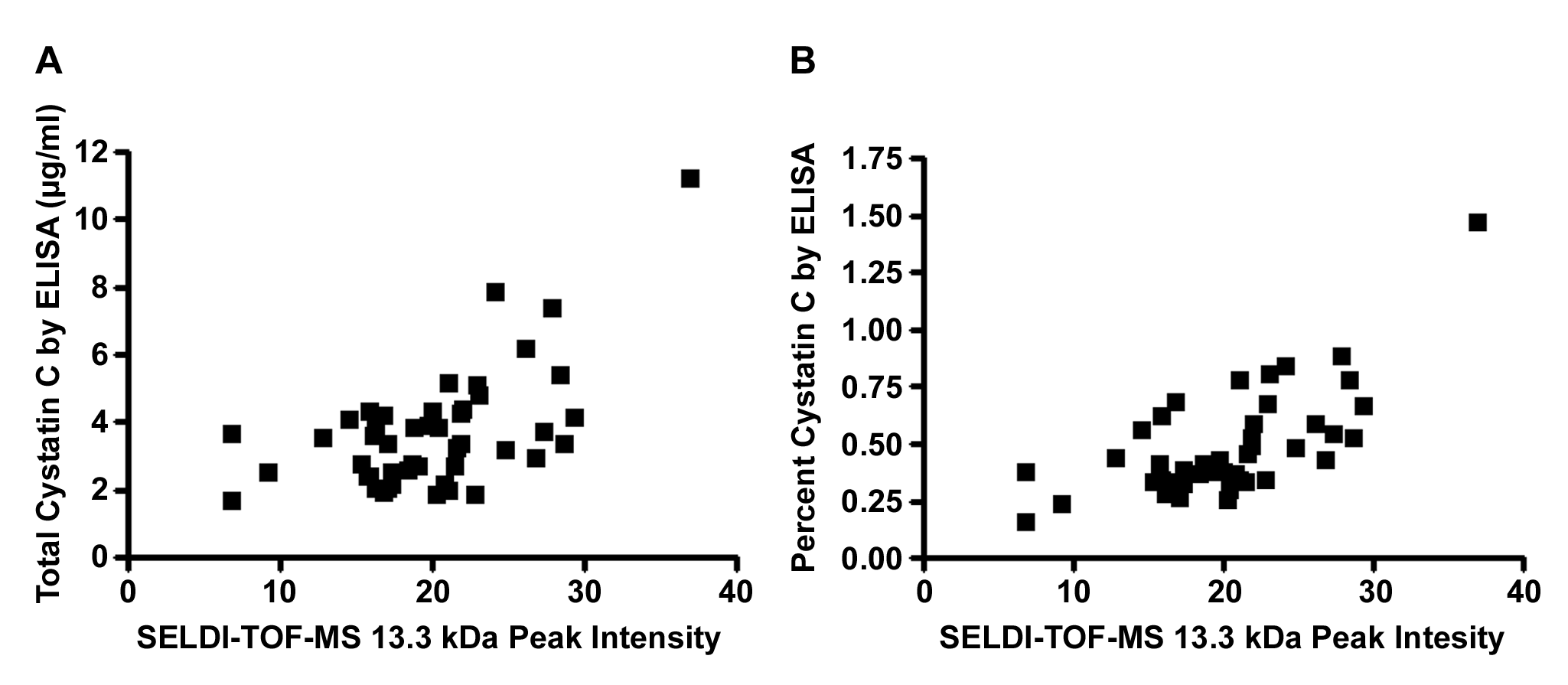

Supplement: Figure S1 — Correlation of ELISA-based cystatin C levels and SELDI-TOF-MS 13.3 kDa mass peak intensity levels by Spearman correlation analysis. Both total (A) and percent (B) cystatin C ELISA measurements correlated to the 13.3 kDa cystatin C mass peak. (TIF) [file pone.0015133.s001.tif]

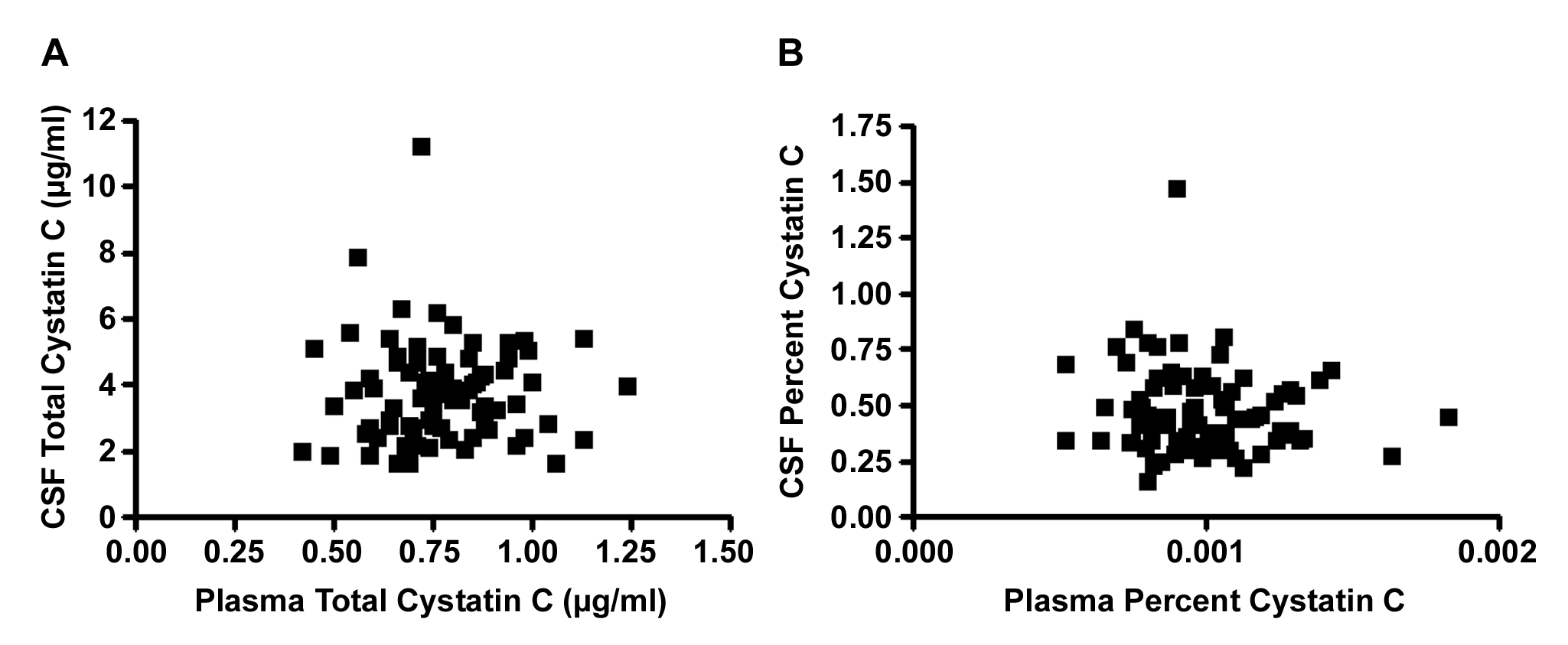

Supplement: Figure S2 — Correlation analysis for cystatin C levels in CSF and plasma. There was no correlation between total cystatin C concentrations (A) (r = 0.055; p = 0.626) or percent cystatin C levels (B) (r = -0.076; p = 0.501) between CSF and plasma. (TIF) [file pone.0015133.s002.tif]
